# Single-cell technologies and spatial transcriptomics: decoding immune low - response states in endometrial cancer

**DOI:** 10.3389/fimmu.2025.1636483

**Published:** 2025-07-02

**Authors:** Yumeng Li, Hua Qiu, Zhenzhen Zhao, Fanghua Qi, Pingping Cai

**Affiliations:** ^1^ The First Clinical Medical College of Shandong University of Traditional Chinese Medicine, Jinan, China; ^2^ Gynecology, Jinan Municipal Hospital of Traditional Chinese Medicine, Jinan, China; ^3^ Traditional Chinese Medicine, Shandong Provincial Hospital Affiliated with Shandong First Medical University, Jinan, China

**Keywords:** single-cell sequencing, spatial transcriptomics, tumor microenvironment (TME), immunotherapy, endometrial cancer

## Abstract

Globally, endometrial cancer continues to impact a significant number of women. Immunotherapy provides those suffering from advanced or relapsed disease hope, but an important barrier is still the absence of trustworthy predictive biomarkers. To tackle this challenge, single-cell sequencing and spatial transcriptomics (ST) are increasingly applied. In cervical cancers of the no specific molecular profile (NSMP) subtype accompanied by p53 mutations. In many cases, the tumor microenvironment (TME) in endometrial cancer exhibits strong immunosuppression or poor immune cell infiltration, often leading to worse clinical outcomes. Single-cell sequencing reveals cellular heterogeneity and helps identify potential therapeutic targets and predict treatment responses. Conversely, ST assists in determining biomarkers that influence the effectiveness of immunotherapy by capturing the spatial organization of tumors. When combined, these technologies allow for integrated multi-omics analysis that aids in the development of immunotherapies, prognostication, and diagnosis. But there are still moral and legal issues. Clinicians may be able to improve outcomes for patients who don’t respond well to current immunotherapies by utilizing these combined approaches.

## Graphical Abstract

The graphical abstract effectively encapsulates the key points of the entire mini-review, offering a concise visual summary of its core content.

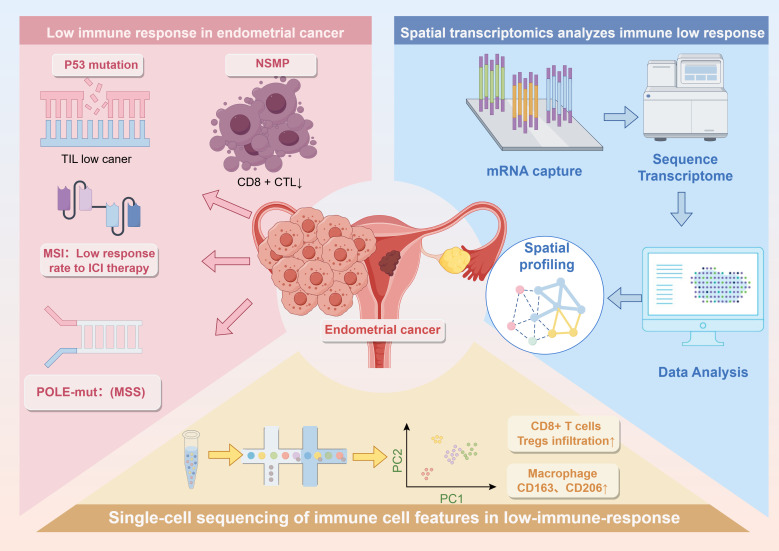

## 1 Introduction

Endometrial cancer (EC), also known as uterine corpus cancer, is a malignant epithelial tumor originating from the endometrial lining. It ranks among the three most prevalent gynecologic malignancies in women. This cancer primarily occurs in perimenopausal and postmenopausal women, with its incidence showing a rising trend in recent years. Based on pathogenesis and biological behavior, EC is classified into estrogen-dependent (Type I) and non-estrogen-dependent (Type II) subtypes (1, 2). Menopausal hormonal shifts and the gradual loss of reproductive function contribute to its development. Vaginal bleeding after menopause affects approximately 90% of patients. Early signs show as minimal serous or bloody discharge, while advanced disease features foul, pus-like fluid (3, 4). Patients often experience dull lower abdominal discomfort, and in late stages, pain in the lower limbs or sacral region may occur. Moreover, an enlarged uterus may be palpable in the lower abdomen of advanced cases (5).

Multiple risk factors contribute to EC progression, including hormonal and reproductive issues like anovulatory menstruation and polycystic ovary syndrome. Additionally, obesity, hypertension, and diabetes significantly increase risk. Infertility also raises susceptibility. Furthermore, prolonged unopposed estrogen therapy further elevates risk. Genetic predisposition plays a role, with about 20% of cases showing family history (6). Endometrial biopsy remains the gold standard for diagnosis. Meanwhile, hysteroscopy allows direct visualization of intrauterine lesions and facilitates targeted biopsy (7).

The primary treatment involves surgery, usually total hysterectomy combined with bilateral salpingo-oophorectomy. This is often complemented by radiotherapy. Chemotherapy is applied for recurrent or advanced disease. Hormonal therapy mainly targets well-differentiated endometrioid adenocarcinoma (8). The prognosis of EC closely correlates with the disease stage. While patients diagnosed at early stages (Stage I and II) achieve a 5-year survival rate between 70% and 95%, those with advanced-stage disease (Stage III and IV) face significantly lower survival rates (9). However, traditional treatments like surgery, radiotherapy, and chemotherapy often show limited efficacy or lead to resistance in certain patients.

Immunotherapy offers new hope for patients with advanced or recurrent EC. Recent studies, such as the NRG-GY018 and RUBY trials, demonstrate that combining first-line chemotherapy with immune checkpoint inhibitors significantly improves prognosis and increases survival rates (10). Compared with traditional chemotherapy, immunotherapy presents a lower incidence of adverse events. Severe complications occur in fewer than 10% of cases, and treatment discontinuation due to toxicity is uncommon (11). Consequently, immunotherapy provides a promising alternative, particularly for those with microsatellite instability (MSI) or mismatch repair-deficient (dMMR) tumors. In such cases, checkpoint inhibitors—whether used alone or in combination—achieve better clinical responses (12). Moreover, the advancement of molecular diagnostic technologies facilitates precise classification of EC, enabling personalized treatment strategies based on genetic features such as POLE mutations or MSI-H/dMMR status (13, 14).

Immunotherapy still faces hurdles, as biomarkers like PD-L1 and MSI-H/dMMR lack consistent accuracy due to biological variability. These limitations reduce their value in guiding treatment. Notably, the NRG-GY018 trial shows that even pMMR patients with low PD-L1 levels benefit from immunotherapy, with a 56% drop in progression risk, suggesting PD-L1 is not a definitive predictor (15, 16). Moreover, significant inter-tumor and inter-patient heterogeneity in biomarker expression and function reduces the predictive value of single markers. Biomarker profiles also change during tumor evolution and treatment, making their predictive power unstable over time (17). Despite its success, immunotherapy faces challenges such as resistance, limited monitoring, high costs, and lack of long-term data. However, single-cell and spatial transcriptomic (ST) technologies help address these issues by uncovering resistance pathways, identifying new targets, and predicting treatment outcomes more precisely (18). On the other hand, ST enables the evaluation of immune responses, the characterization of the tumor microenvironment, and integration with imaging to inform personalized medicine, ultimately reducing toxicity and increasing treatment effectiveness. **Graphical Abstract** presents the graphical abstract of this paper.

## 2 Clinical features of immune-nonresponsive EC

### 2.1 Clinicopathological characteristics of immune-low EC patients

From a molecular classification perspective, p53-mutated EC is categorized as an immunosuppressive subtype. This group typically displays elevated levels of Tregs, M2 macrophages, PD-L1^+^CD68^+^ macrophages, and CD8^+^PD-1^+^ T cells, which correlate with the poorest survival outcomes. In contrast, the NSMP subtype of EC demonstrates an immune-desert phenotype, with minimal infiltration by immune cells such as CD8^+^ cytotoxic T lymphocytes and low expression of immune checkpoint molecules. Although generally well-differentiated, NSMP tumors also exhibit high levels of tumor-associated macrophages (TAMs) and are associated with unfavorable prognoses (19–21).

Various stromal and immune-related cells within the tumor microenvironment contribute to immunosuppression and tumor progression. Cancer-associated fibroblasts secrete cytokines such as TGF-β and IL-6, which suppress immune responses and restrict immune cell infiltration (22). Tumor-associated neutrophils, particularly the N2 subtype, promote tumor growth and impair cytotoxic T cell activity (23). Additionally, mesenchymal stromal cells modulate immune function through paracrine signaling, further enhancing immunosuppression within the tumor niche (24).

Additionally, low immunogenicity correlates with hormone receptor status. The absence of both ER and PR expression independently predicts poor prognosis in EC, even among low-grade tumors. Specifically, ER-negative tumors tend to have reduced FOXP3^+^ Treg infiltration, while PR-negative cases show increased TAM presence, potentially promoting immune tolerance and worse outcomes (25, 26).

The tumor immune microenvironment in immune-nonresponsive EC is typically characterized by dysfunctional or sparse immune infiltration. While p53-mutated tumors exhibit features of an immunosuppressive microenvironment, NSMP tumors generally lack a competent anti-tumor immune response (27).

### 2.2 Clinical outcomes and prognostic implications of low immune response

In EC, immune checkpoint blockade proves less effective for about 70% of MSS or pMMR cases. The KEYNOTE-028 study supports this, reporting just 13% response and 13% disease stabilization with pembrolizumab (28, 29). Patients with immune-nonresponsive EC generally have a poor prognosis. Specifically, p53-mutated EC is associated with the lowest survival due to its immunosuppressive microenvironment. Likewise, NSMP EC also demonstrates poor clinical outcomes, likely due to ineffective antitumor immune responses (30, 31).

### 2.3 Current clinical exploration of biomarkers for immune-low EC

Among immune cell-associated markers, CXCL8_hi_ IL1B_hi_ macrophages have been identified as predictors of poor and non-durable responses to ICIs in EC. A high-risk score derived from six associated genes correlates with shorter survival and decreased ICI efficacy. Moreover, the presence of dysfunctional (CD8^+^PD-1^+^) or terminally exhausted (CD8^+^PD-1^+^TOX^+^) T cells, along with their interactions with PD-L1^+^ cells, independently predicts 24-month progression-free survival (PFS) in dMMR uterine or ovarian cancer patients receiving nivolumab (32, 33). In the context of the tumor immune microenvironment, TIL-low tumors—often seen in ECs with either mutant or wild-type p53—lack distinct immunologic features. Though they may also appear in MMR-deficient or POLE-mutated tumors, these TIL-low tumors generally indicate an immune-cold phenotype (34). Additionally, molecules linked to immunosuppression, such as PD-L1, may be related to poor immune reactivity in EC, although further studies are needed to confirm this association.

## 3 Harnessing single-cell and ST for EC management

Single-cell sequencing provides a powerful tool for understanding the complexity of EC. It uncovers intratumoral heterogeneity, identifies discrete cell populations, and defines their transcriptomic signatures, which collectively enhance the understanding of tumor biology and support personalized treatment development (35, 36).

Additionally, it helps detect novel therapeutic targets that are often hidden in bulk sequencing data, such as subgroup-specific receptors and signaling pathways, paving the way for novel drug development (37). It also offers dynamic insights into gene expression changes induced by treatment and uncovers variable drug sensitivities among cell types, enabling clinicians to anticipate treatment responses and adapt strategies in advance. Moreover, by revealing immune cell diversity, function, and interactions within the tumor microenvironment, it contributes to understanding immune resistance and tailoring immunotherapy (38).

Conversely, ST combines gene expression analysis with spatial information from histological sections. It characterizes the spatial distribution of cell populations and their roles within tumors and their adjacent microenvironments (39, 40). With such detailed data, it becomes possible to better define tumor subtypes and assess malignancy levels, guiding therapeutic planning in terms of surgical extent, radiation dosage, and chemotherapy selection (41). In addition, this method elucidates immune gene expression and immune-tumor spatial relationships, facilitating the identification of immunotherapy response biomarkers. ST also helps visualize drug localization and effects, improving therapeutic targeting, reducing adverse effects, and informing rational clinical trial design (42–45).

## 4 Application of single-cell technologies in clinical immunological research of EC

### 4.1 Workflow and challenges of single-cell sequencing in clinical samples

The main workflow involves sample processing and cell preparation. After obtaining EC tissues and matched adjacent normal tissues, they must be dissociated into single-cell suspensions. This step aims to maintain cell viability and integrity while minimizing cell death and contamination. Due to the high heterogeneity of tumor tissues, it is essential to ensure that all cell types are represented in the suspension (38). For single-cell RNA sequencing, the 10x Genomics Chromium platform offers high throughput at a relatively low cost. Yet, it requires careful experimental handling and involves intricate operations. Additionally, variations among platforms lead to inconsistent data, complicating cross-study comparisons (46). Data analysis includes quality control, normalization, dimensionality reduction, and clustering, relying on diverse bioinformatics tools and algorithms. Cell type annotation depends on known marker genes, but the limited availability of markers hinders comprehensive and accurate identification. Additionally, data analysis demands significant computational resources and expertise, with workload increasing exponentially as experiment size grows (47). Integrating and sharing data across studies can improve accuracy, but differences in experimental conditions, sequencing platforms, and analysis methods pose challenges. Therefore, more effective integration methods and standardized workflows are needed, along with data sharing platforms to promote communication and accelerate EC research.

### 4.2 Analysis of immune cell features in patients with low immune responsiveness

Single-cell sequencing reveals immune cell changes associated with EC treatment resistance. Notably, exhausted CD8+ T cells increase, expressing more inhibitory receptors that reduce anti-tumor activity. Meanwhile, regulatory T cells (Tregs) and myeloid-derived suppressor cells (MDSCs) also rise, creating an immunosuppressive environment that promotes resistance to immunotherapy (48–50). Potential biomarkers for evaluating immune cell status and forecasting immunotherapy response include molecular indicators of immune cell dysfunction, such as improved levels of the exhaustion markers PD-1, CTLA-4, and LAG-3 on T cells, as well as M2 macrophage polarization markers CD163 and CD206 (51). By analyzing immune cell infiltration, functional states, and intercellular interactions in the tumor microenvironment (TME) through single-cell data, researchers identify potential immunotherapy targets. For example, therapies targeting molecules involved in immune dysfunction or drugs promoting macrophage polarization toward the M1 phenotype enhance anti-tumor immunity (52). Furthermore, integrating clinical features and treatment responses helps screen targets related to therapy efficacy, thereby improving clinical trial success rates (53). Standard endpoints such as survival and tumor reduction may not accurately represent early immunotherapy effects. Alternatively, single-cell approaches detect immune cell dynamics and functional shifts in the TME, enabling early response evaluation and optimized care (54).

## 5 The value of ST in clinical practice of EC

### 5.1 Examples of spatial transcriptomic applications in clinical tissue

ST integrates into early clinical trials for EC. For instance, in a trial targeting the Netrin-1 antibody (NP137), researchers collect biopsy samples and analyze them, revealing that the treatment reduces the expression of genes related to epithelial-mesenchymal transition (EMT). This finding not only offers new insights for clinical therapy but also supports subsequent drug development and clinical trial design (39). In research on high-grade serous ovarian cancer, spatial analysis comparing tumors from long-term and short-term survivors shows that tumors from long-term survivors contain a more diverse infiltration of immune cells. This observation provides a reference for studying the immune microenvironment in EC and helps clarify the role of immune cells in tumor progression (55).

### 5.2 Building spatial maps of the immune microenvironment with clinical significance

ST reveals detailed immune microenvironment profiles in EC at various phases. Immune cell infiltration and spatial distribution, along with intercellular interactions, undergo significant changes as the tumor advances. Early-stage tumors show localized immune presence, while later stages have widespread immune infiltration and enriched suppressive cells, which influence therapeutic results and prognosis (56). In addition, the immune landscapes of primary tumors and metastatic lesions present notable differences. ST studies indicate that immune cell composition and activity in metastatic sites may not mirror those in primary tumors. Some immune subtypes may be more active in metastases, or the interactions between immune and tumor cells may shift. Understanding these variations is important for deciphering metastasis and formulating precise therapeutic strategies (57, 58).

### 5.3 Spatial information guides precise immunotherapy decisions

The development of tailored immunotherapy approaches is made possible by the spatial analysis of the immune microenvironment within individual tumors. For instance, combining immune checkpoint inhibitors with medications that target immunosuppressive cells may be taken into consideration if a tumor has a high concentration of these cells. Conversely, when immune cell infiltration appears limited, it becomes essential to explore strategies aimed at enhancing immune cell activation and recruitment (58). Spatial data helps identify exact targets and synergistic actions of drugs in combination immunotherapies. For instance, it guides whether immune checkpoint inhibitors boost immune cell activity and if chemotherapy enhances immune infiltration by analyzing the spatial layout of immune and tumor cells within the TME (59).

## 6 Clinical translation of combined single-cell and ST in immunotherapy for EC

### 6.1 Multi-omics integration aids clinical diagnosis and prognosis

Combining spatial and single-cell transcriptomic technologies reveals both cellular heterogeneity and tissue architecture in EC. This helps develop accurate biomarkers for early diagnosis and guides therapy in advanced or recurrent cases (60). Additionally, multi-omics profiles enable precise risk classification and treatment stratification, which serve as the foundation for personalized therapeutic strategies. Early identification of high-risk patients permits proactive monitoring and intervention to minimize relapse, and specific therapies for distinct risk groups improve treatment efficacy and patient survival.

### 6.2 Promoting the development of novel immunotherapeutic drugs

The combined use of single-cell and ST technologies enables a deep understanding of the immune microenvironment and molecular mechanisms of tumor cells in EC, providing a theoretical foundation for designing new immunotherapies. For instance, identifying the critical roles of TAMs and MDSCs in tumor immune evasion offers potential targets for developing immune-modulating drugs. Moreover, clinical trials employ single-cell and spatial transcriptomic analyses to monitor and evaluate drug efficacy in real time, while also revalidating drug targets to ensure the direction and effectiveness of drug development. By examining changes in immune cell subsets and functions before and after treatment, researchers assess immunomodulatory effects, which guide drug optimization and improvement.

### 6.3 Ethical and regulatory considerations in clinical translation

In the clinical application of single-cell and ST technologies, strict adherence to relevant laws and regulations is essential. Moreover, protecting patient privacy and managing data security are critical to prevent unauthorized access or misuse of personal and genetic information. Techniques such as anonymization and encryption help safeguard privacy, while robust data security systems prevent breaches and illegal use. Furthermore, following rigorous clinical trial and regulatory protocols ensures the safety and efficacy of these new technologies, thereby protecting patients’ health and rights. Before clinical implementation, comprehensive preclinical studies and clinical trials must verify their safety and effectiveness, and approval from regulatory authorities is required in accordance with applicable laws.

## 7 Discussion

The TME in immune-low EC patients shows abnormal immune cell infiltration and impaired function (61). The immune infiltration pattern in p53-mutant EC suggests a strong immunosuppressive microenvironment, whereas the NSMP subtype lacks an effective antitumor immune response. For instance, TIL-low tumors generally lack immunological features and are more common in tumors with p53 abnormalities or wild-type p53, indicating an immune low-responsive state (62, 63). Immune low responsiveness allows tumor cells to evade immune surveillance, proliferate extensively, and develop into malignancies. It also alters the TME, accelerating tumor growth and metastasis while reducing patient survival and cure rates (64). In treatments that rely on the patient’s immune system, such as cancer immunotherapy and anti-infection immunotherapy, immune low responsiveness significantly diminishes therapeutic efficacy (65). Exploring immune low responsiveness reveals underlying disease mechanisms and immune impairments involving immune cell changes and signaling disruptions. This insight offers a foundation for clinical diagnosis, treatment, and prevention and facilitates the discovery of biomarkers and targets for new diagnostic tools and therapies (66) A thorough understanding of immune low responsiveness contributes to better immunotherapy outcomes, facilitates individualized treatment planning, and improves overall patient care. Furthermore, investigating its underlying causes and risk factors promotes early action to lower disease rates, refine vaccine strategies, and elevate vaccine-induced immunity. This knowledge also helps delay immune aging and informs preventative measures to safeguard older populations from age-associated illnesses (67).

Single-cell transcriptomics offers a precise method to capture cellular heterogeneity by profiling individual cells’ gene expression. This technique identifies distinct cell subsets and their functional statuses, enabling the detection of rare and low-abundance cells contributing to immune hypo-responsiveness. By systematically examining immune cell characteristics, it identifies key states such as activation, exhaustion, and regulation, while also clarifying associated molecular pathways. In addition, through marker-based and gene expression analysis, it anticipates intercellular communication and, using computational algorithms, models spatial structure with reasonable accuracy (68). Despite its advantages, single-cell RNA-seq cannot determine precise spatial arrangements of cells or their microenvironmental influences. Moreover, technical challenges and high costs make it less feasible for large-scale research (56). In contrast, spatial transcriptomics captures gene expression within intact tissues, preserving spatial context. It reveals immune infiltration patterns and tumor-immune interactions while highlighting tissue structure and cell signaling. When integrated with single-cell data, it enhances biological interpretation (69). However, its resolution is often insufficient to precisely separate cell types or identify rare cells. The demanding sample processing can reduce capture efficiency and data integrity. Furthermore, its data analysis requires complex algorithms and high computational expertise (70).

In upcoming research, combining Mendelian randomization may provide a more detailed understanding of the metabolic and microbiota-associated pathways involved in endometrial cancer (71–74). Additionally, incorporating nanotechnological advances and single-cell genomics could inspire new therapeutic directions (75–77). At the same time, focusing on the role of immunodynamics in endometrial cancer (29) and developing predictive models (78–80) may lead to improved clinical decision-making.

## 8 Conclusion

The analysis of clinicopathological features in patients with immune hypo-responsiveness provides new perspectives and directions for precision treatment of EC. By integrating multi-omics data, comprehensive biomarker models are developed to enable precise risk stratification and therapeutic grouping, which also facilitates the development of novel immunotherapies. This approach holds promise to improve outcomes for patients exhibiting immune hypo-responsiveness. Meanwhile, ethical and regulatory considerations must be thoroughly addressed during clinical translation to ensure that these technologies remain safe, effective, and sustainable.
